# Predicting Freezing Point of Ice Cream Mix with Infrared Spectroscopy and Chemometrics

**DOI:** 10.3390/foods15142549

**Published:** 2026-07-19

**Authors:** Duy Thinh Trinh, David Mcintosh, Elizabeth Eckelkamp, Jiajia Chen, Alejandro Molina-Moctezuma, Tong Wang

**Affiliations:** 1 Department of Food Science, University of Tennessee, Knoxville, TN 37996, USA; 2 Department of Plant Sciences, University of Tennessee, Knoxville, TN 37996, USA; dmcintos@utk.edu; 3 Department of Animal Science, University of Tennessee, Knoxville, TN 37996, USA; 4 School of Natural Resources, University of Tennessee, Knoxville, TN 37996, USA

**Keywords:** infrared spectroscopy, prediction, modeling, freezing point, ice cream mix, FTIR

## Abstract

Fourier transform infrared spectroscopy (FTIR) has become an increasingly valuable analytical tool in the dairy industry due to its rapid, non-destructive nature and ability to capture complex chemical information. This study evaluated the feasibility of using FTIR in combination with chemometric modeling to predict the freezing point (FP) of ice cream mix (ICM), an important quality attribute that influences product texture, freezing behavior, and manufacturing efficiency. Traditional methods for FP determination are often labor-intensive and can present challenges when analyzing high-solids dairy systems. In this study, FP values were obtained through a cryoscopic method. FTIR spectra were collected for all samples, and partial least squares (PLS) regression models were developed to relate spectral information to FP values. Model performance metrics indicated that FTIR spectra contained information associated with FP variation; however, overall predictive performance was limited. The relatively weak model accuracy was attributed to the complex nature of FP as a colligative property influenced by multiple compositional factors, including sugars, salts, proteins, and stabilizers, as well as the narrow compositional range and limited sample size of the dataset. Despite these limitations, the results demonstrated the potential of FTIR as a rapid screening tool for FP estimation and provided a foundation for future model development using larger and more compositionally diverse datasets and improved reference methodologies.

## 1. Introduction

Ice cream is the most popular dairy product in the world. With more than $11.6 billion of direct economic impact to the U.S. economy as of 2024, ice cream products play a major role in the dairy industry [1]. In ice cream, the required amount of milk solids is not less than 20%, and the required amount of milk fat is not less than 10% [2]. The determination of ice cream mix (ICM) freezing point (FP) is important in the industry as it directly relates to formulations and quality. Manufacturers strive to achieve low production costs, while maintaining the quality and shelf-life of products. The FP of ICM depends on the soluble components in the mixture and varies based on its composition [3]. Sugars are the primary component that lowers the FP, while milk solids non-fat (SNF) or added whey solids have a smaller impact. There is a need to rapidly and accurately quantify ICM FP in the industry. As FP is used as a quality parameter, ICMs with lower levels of fat or protein may have their FP behavior altered using stabilizers, sweeteners, or other additives, making accurate FP determination essential for verifying formulation and performance integrity. Because ICM formulations vary widely in fat, protein, sugar, total solids (TS), SNF content, and additives, and because freezing point is a key quality-control parameter that governs texture, freezing efficiency, and product consistency, a robust and rapid method for determining FP is essential for managing formulation variability, optimizing processing conditions, and maintaining consistent product quality across production runs.

Infrared spectroscopy (IR) has been widely used to determine FP of dairy products. Prediction databases and models for FP of dairy, especially milk, have been constantly updated, validated, and evaluated for accuracy and precision with the purpose of detection of adulteration [4,5,6,7,8,9,10,11]. IR is a technology that analyzes the interaction of molecules with light in the wavelength region of infrared (0.7–1000 µm). Chemometric techniques are used to analyze spectra and create predictive calibration models based on reference chemistry data. Regular validation and calibration of models are required for these prediction models to be accurate [12]. IR is valued for quality control and analytical scanning due to its speed, accuracy, and non-destructive nature, offering advantages over traditional analytical chemistry methods. However, in dairy products, FP is not related to a single component that can be detected through IR signals, but rather to a combination of all the solid components (fat, protein, lactose, salts, and minerals) in the matrix [8,13,14,15]. Cryoscopy has been the industry standard for measuring FP of milk and dairy products [16,17,18]. Although being widely adapted for dairy applications, IR calibration development is not yet established for ICM. The existing prediction model for ICM on the FOSS MilkoScan™ FT3 is based on frozen dessert products and is considered a clone. There is no database for ICM within the available FOSS calibration library (FOSS, Hilleroed, Denmark).

Cryoscopy is a standard scientific method of determining the FP of a liquid and is often used to analyze the effect of dissolved substances on the FP. This technique is based on the principle of FP depression, where the addition of solutes lowers the freezing temperature of a solvent [18]. Cryoscopy has practical applications in the dairy industry to measure the FP of milk for detecting adulteration, or in chemistry, to determine molecular weights of solutes in solutions [19,20,21,22,23]. While fast and accurate, the use of the cryoscope is limited and exclusively calibrated to milk and other liquids with low concentrations of solids. In such products, sugar is the main influencer on FP depression, where higher concentration of sugar results in higher FP depression, posing difficulties for the instrument to freeze the sample [24,25,26,27]. For the scope of this study, FP of ICM was determined by extrapolation from FP using a series of dilutions of the ICM.

In this study, ICM samples were subjected to spectral scanning using FTIR and cryoscopic measurements for FP. Data from FTIR and cryoscope were used to establish a quantitative calibration model, which is not yet readily available for industrial use in FOSS systems. The specific objective of this work is to prove the concept before starting a much larger set of samples for industrial scale modeling.

## 2. Materials and Methods

### 2.1. Sample Selection

ICMs were generously provided by Mayfield Dairy Farms^®^ manufacturing facility (Athens, TN, USA), with samples collected directly from individual production batches. The final number of samples and ICM type depended on production availability at the facility, totaling 43 samples. Collected samples had fat levels of 12%, 10%, and 5%, with 10% being the most heavily weighted due to its higher production frequency at the facility. Details on the number of samples at each fat content level are listed in Table 1. While sample collection was initially categorized solely by these fat tiers, as no other technical specifications were provided by the manufacturer at the time of collection, full compositional parameters (including protein, lactose total solids, and solids non-fat) were subsequently analyzed for each sample using the calibrated FOSS system to evaluate their broader impact on freezing point behavior. The use of these parameters is described in Section 2.4 below.

After model generation, an external set of 5 samples with various fat levels (1 at 12%, 3 at 10%, and 1 at 5%) was used for external validation of the established model.

### 2.2. Modifying Cryoscopy Method for Freezing Point Determination of Ice Cream Mixes

The cryoscope AI 4250 (Advanced Instruments, Norwood, MA, USA) was used to determine FP of ICM. Due to the parameters of the cryoscope, which were set up for milk, ICMs were unable to freeze in the cryoscope. Because sugars, macromolecules, and additives were the main factors impacting the FPs, and their concentrations were much higher compared to milk, each ICM had to be subjected to serial dilution to achieve 50%, 25%, 12.5%, and 6.25% (*v*/*v*) ICM content with deionized water. An aliquot of 2.5 mL was taken from each dilution to measure the FP. FP was plotted against percent ICM to achieve a linear curve, and extrapolation was used to determine the FP of the original ICM.

### 2.3. Spectral Collection Using FTIR

Samples were scanned using MilkoScan™ FT3 (FOSS, Hilleroed, Denmark) for spectral data. ICMs were homogenized and measured directly to obtain spectra. Measurement time for each sample was less than 10 s, and the wavelength range was 2.5 to 25 µm.

### 2.4. Statistical Exploration Using R to Determine the Impact of Composition of FP

Data for FP collected from cryoscopy and ICM composition from the cloned FOSS prediction models were used to establish linear multiple regression models to assess the impact of protein, fat, lactose, TS, and SNF on FP (FOSS, Hilleroed, Denmark). Since FOSS model predictions for these components were accurate with R^2^ of 0.99 (FOSS Application Note AN 5485 Rev. 2 for the MilkoScan™ FT3), these compositional data were used for relationship observation. Models were validated with a 5-fold split (4 for training and 1 for validation) and checked for linear model assumptions and predictor selection based on R^2^, akaike information criterion (AIC) values, and variance inflation factor (VIF). All statistical modeling was performed using R (2024.12.0 Build 467) [28]. The following two packages were used: car (3.1.3) [29] and AICcmodavg (2.3.3) [30].

### 2.5. Calibration Model Generation

**Figure 1:**
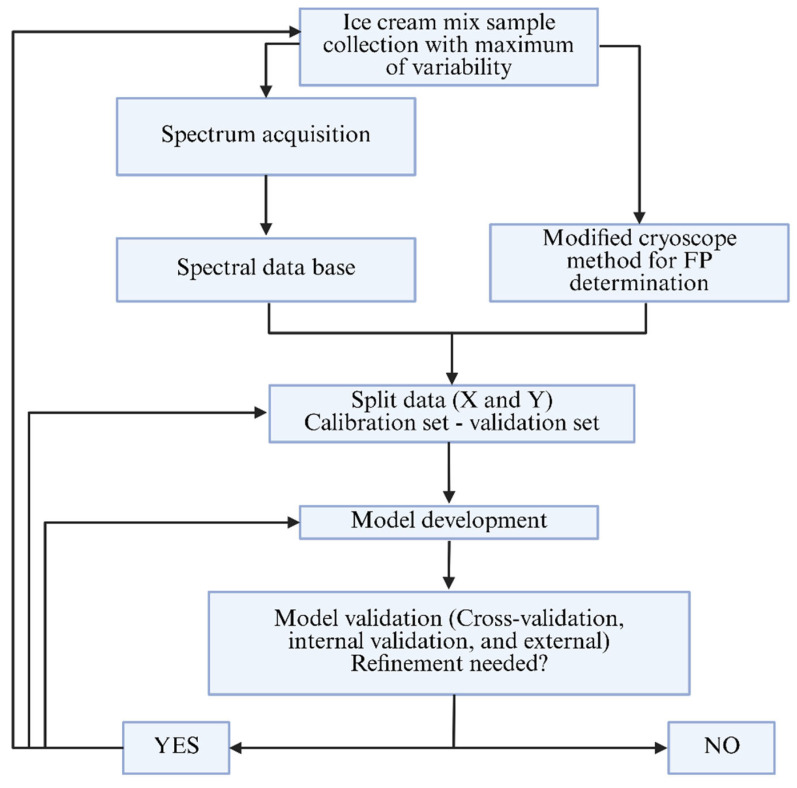
Logic flow of model generation for determining ice cream mix’s freezing point.

A quantitative calibration model for FP was generated using FOSS Calibrator Pro Software 3.9.2.10 (FOSS, Hilleroed, Denmark). Briefly, PLS method was implemented for correlation between ICM FP and spectral data, where 80% (103) of the total data points were randomly selected for a training set and the remaining 20% (26) for internal validation. Root means square error of prediction (RMSEP) and R^2^ were reported for both training and validation sets of each model and were used to select models. The proposed logic flow of model generation is described in Figure 1. The following information was collected: mean of the reference data, SD of the reference data, method of developing calibration model, method of calibration evaluation, number of samples used for calibration and testing, mathematical data pretreatment, number of factors, standard error of calibration, and standard error of cross-validation [31].

### 2.6. The Impact of Sugar and Protein in Ice Cream Mix on Freezing Point Measurement

Since correlation analysis () indicated that sugars and protein had significant impact on FP, ICM model systems were created using commercially available non-fat dry milk powder containing 34.78% protein and 52.17% lactose (dry weight basis) to evaluate this impact. The ICM was based on regular commercial ice cream with protein content of 4% (*w*/*w*) and sugar content of 22.67% (*w*/*w*). A stock solution of ICM was made by dispersing the non-fat dry milk powder in deionized water at 11.5% (*w*/*v*) and letting it hydrate for 12 h. From the stock dispersion, three ICM matrices were made, each containing a single added sweetener: lactose, sucrose, or corn syrup (33.33% (*w*/*v*) glucose, 66.67% (*w*/*v*) oligosaccharides). For each ICM matrix, the selected sweetener was added to the final sugar content of 22.67% (*w*/*v*), including the 6% (*w*/*v*) lactose content in the stock ICM dispersion. A second set of three samples was prepared using only deionized water and the individual sweeteners (lactose, sucrose, or corn syrup), without the addition of non-fat dry milk powder to determine the effect of sugar alone on freezing point and eliminate any sugar–protein interactions. Each sample matrix was determined for FP using the modified cryoscopy method as described above.

## 3. Results and Discussion

### 3.1. A Modified Cryoscopy Method for Freezing Point Determination of Ice Cream Mixes

The cryoscope AI 4250 was a reliable quality control instrument for milk in the dairy industry. However, the instrument was unable to determine the FP of ICM. Due to the higher concentrations of sugars and fats (more than 20% *w*/*v*), samples did not freeze in the preliminary trial with Sysco ICM. According to Blagden’s law on FP depression, the FP of a solution is inversely proportional to the concentration of the solution, meaning the more concentrated the solution, the lower the FP [32]. This was the rationale for diluting ICMs for plotting and extrapolation to determine the true FP of the ICM.

**Figure 2:**
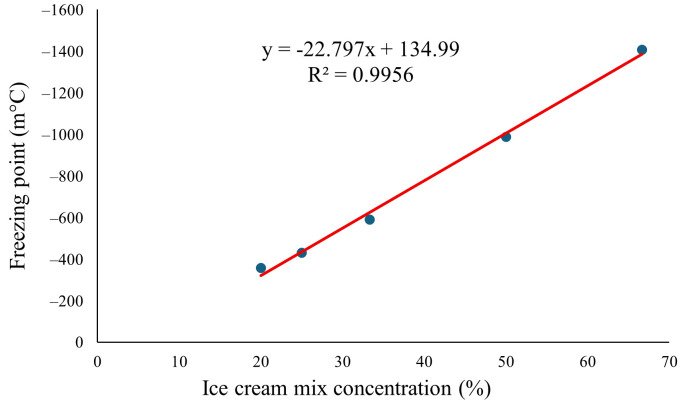
Freezing point determined based on its relationship with concentration vs. concentration of ice cream mix (Sysco) in the preliminary study, with R^2^ of 0.99.

A preliminary trial was conducted on Sysco ICM to determine the feasibility of this method. A linear relationship with R^2^ of 0.99 was observed for this sample (Figure 2). The extrapolated FP of this sample was −2145 m°C (i.e., milli degrees centigrade).

**Figure 3:**
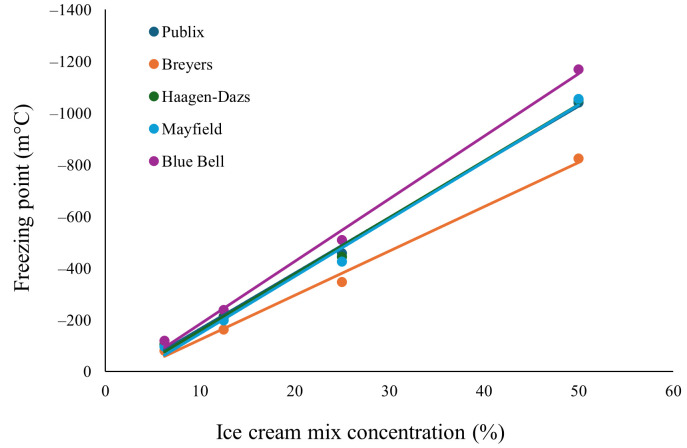
Freezing point of commercial ice cream vs. ice cream mix concentration from cryoscopy extrapolation (R^2^ of 0.99 for all ice cream mixes). Different ice cream brands have different freezing point–concentration effects.

Figure 3 shows the linear relationships between commercial ice cream mix dilutions and FPs. Regression equations were used to extrapolate the FP of the undiluted ICMs, and FPs from extrapolation are shown in Table 2. The matrix effect was also observed. The difference in slopes of different ice cream products indicates that the base formulation could significantly impact FP. Each type of sweetener used in formulation can have a different effect on FP depression [27]. In a sample matrix, the effects of individual sweeteners are influenced by the matrix effects. From these findings, this modified cryoscopic method was found to be suitable to determine FP of ICM.

### 3.2. Freezing Point of Ice Cream Mixes Determined Using the Modified Cryoscopy Method

FPs of ICMs are presented in Table 3, with FP of ICM spanning from −2355 to −1933 m°C. The coefficient of variation (CV) values represent variations in the same product across batches.

### 3.3. Influence of ICM Components on FP as Determined by Statistical Exploration

Multiple linear regression models were fitted to the compositional dataset generated from the existing cloned model to explore the correlation between FP and other components (fat, protein, lactose, SNF, lactose, and lactic acid) with predictions for these components that were based on cream. The linear model was implemented by the equation below:

$$y_{i}=b_{0}+b_{1}x_{i1}+b_{2}x_{i2}+b_{3}x_{i3}+\ldots +b_{n}x_{in}+\epsilon_{i}$$

where $y_{i}$ is the response variable (FP); $x_{1}$, $x_{2}$, and $x_{3}$ are the explanatory variables (fat, protein, TS, etc.); $b_{0}$ is the intercept coefficient; and $b_{i}$ are the linear effect coefficients. Table 4 shows fitted models with their R^2^ and AIC values, as well as VIF values of each variable.

The full model was first fitted to the data but did not fit very well. VIF values were very high for fat, TS, and SNF. In linear model, VIFs need to be below 10 to satisfy non-collinearity assumption. The reason is TS is calculated by adding all the solids, including fat, and SNF is calculated by subtracting fat from TS. Sugars are also calculated by adding glucose, sucrose, and lactose. This resulted in multi-collinearity in the model.

A backward stepAIC function was implemented to automatically eliminate variables to improve (or lower) AIC while keeping R^2^ consistent, resulting in model 1, model 2, and model 3. This approach was very useful in variable selection as it was automated and fast. However, this method eliminated variables based on AIC, not VIF, resulting in the presence of variables with high VIF values. Therefore, variables were eliminated manually to avoid multi-collinearity assumption violation, starting with all the variables from the full model, with the sacrifice of R^2^. By removing variables with high VIF, model 4, model 5, and model 6 were generated. From the full model, SNF was removed to create model 3. From this model, sucrose with high VIF was then removed to create model 4. For model 4, all the VIF were below 10, except for fat. Since fat was one of the major components, the effect of fat on FP was of interest. Model 5 (without TS) and model 6 (without fat) were then fitted. The model without fat did better in terms of VIF. Chemically, polymers like fat should not contribute to FP, as fat globules do not truly dissolve in liquid, and FP is expected to only relate to the concentration of the solubilized solids in the solution. Protein, however, can affect FP as they are partially soluble, not to mention the antifreeze properties of some albumin species [33].

The top three models (in terms of AIC) were generated by a stepAIC, but these models had very high VIF values. Model 4 to model 6 did better, with model 6 having the lowest VIF values, indicating no multi-collinearity violation. For this data, without referring to R^2^ of all fitted models (ranging from 0.28 to 0.36), it can be concluded that protein, TS, lactose, glucose, and sugars can best predict the FP. R^2^ values could be improved by implementing data transformations on FP or variables, or by fitting other types of models. For this statistical exploration, these findings are sufficient to determine important factors that influence the FP of ICMs.

**Figure 4:**
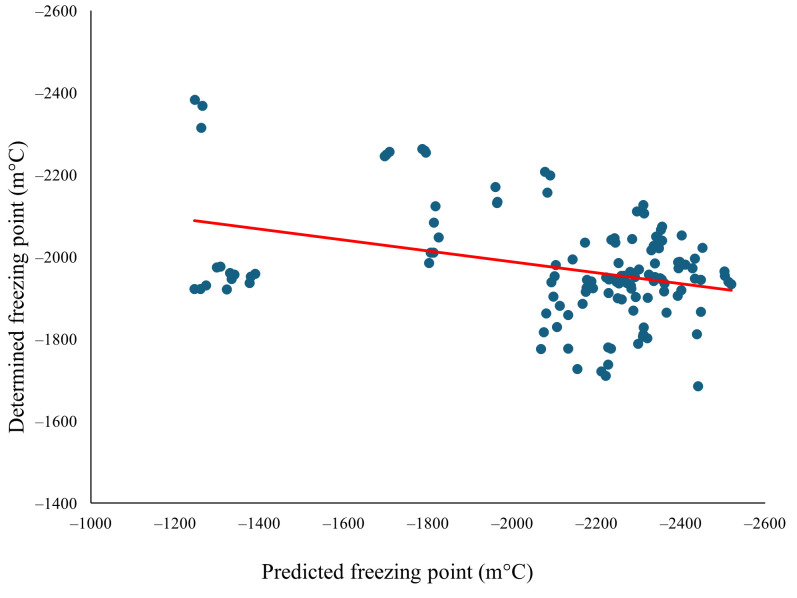
Determined freezing point of ice cream mixes vs. predicted freezing point from cloned model (R^2^ = 0.1232), indicating weak prediction power.

The determined cryoscopic FP data and predicted FP data from the FOSS cloned model also correlated poorly with each other (R^2^ = 0.1163) (Figure 4). This confirmed that the cloned model used for prediction was outdated and a new model needs to be established, hence the justification for this feasibility study.

### 3.4. Calibration Model

A partial least squares (PLS) calibration model was developed to evaluate the feasibility of predicting the FP of ICMs using mid infrared spectra collected on a MilkoScan™ FT3 instrument. The dataset consisted of 103 training samples and 26 internal validation samples. The FP values in the training set ranged from −2207.79 to −1721.44 m°C with a mean of −1947.52 m°C, while the validation set ranged from −2170.07 to −1788.68 m°C with a mean of −1952.47 m°C. The ranges of FP for 12%, 10%, and 5% fat levels were −1950.08 to −1885.37 m°C, −2382.5 to −1710 m°C, and −2022.1 to −1684.9 m°C, respectively. The dataset was heavy on the 10% fat level and did not represent realistic variability.

The model was built using three PLS factors, with no scatter correction and a derivative sequence of [0, 0, 1, 1]. The model retained three spectral wavelength regions after pretreatment: 1150–1538 nm, 1722–1800 nm, and 2700–2954 nm. These regions include absorption bands associated with water, lactose, salts, and other soluble solids, which are components known to influence FP through colligative effects.

The calibration results indicated low predictive ability (Table 5). The training set achieved an RMSEP of 118.229 m°C and an R^2^ of 0.269, meaning that only about 27% of the variance in FP was explained by the model. The slope of 1.232 and intercept of 449.282 indicate a tendency toward overprediction at lower FP values. Bias was low (−7.029 m°C), suggesting minimal systematic error. According to general chemometric standards, R^2^ values below 0.40 indicate poor model strength, which is expected when the analyte is not strongly represented in the spectral domain [34]. Cross-validation with the Venetian Blinds method achieved stronger performance, with an R^2^ of 0.926 and an RMSEP of 119.2 m°C. The large improvement in R^2^ from calibration (0.269) to cross-validation (0.926) suggests that the calibration fit may have been influenced by a few samples, weighted heavily with the 10% fat samples with significantly fewer samples for the 5% and 12% levels, with high leverage or non-linear behavior.

Internal validation with the 26-sample set showed weak performance, with an R^2^ of 0.085 and an RMSEP of 104.7 m°C. The slope of 0.553 indicated that the model did not track changes in FP proportionally to the reference values. These values fall below the ranges typically reported for FTIR prediction of dairy traits, but this is expected given that FP is a colligative property rather than a direct spectral analyte, as well as the smaller number of samples in this proof-of-concept study. For comparison, PLS models for milk fatty acids and cheese yield often achieve R^2^ values of 0.30–0.75 [35], while FTIR prediction of physiological traits in milk typically yields R^2^ values of 0.50–0.85 [36]. Even advanced machine learning models for milk traits often report R^2^ values between 0.20 and 0.80 [37]. These benchmarks highlight that the low R^2^ in the present FP model reflects the inherent difficulty of modeling FP directly from IR spectra rather than a methodological flaw. The validation bias was small (bias = −7.822 m°C), confirming that the model did not systematically shift predictions but struggled with accuracy.

Due to the poor predictive performance of the ICM FP model, the external validation step was not conducted. Further sampling and data collection are needed to build a robust database for an accurate prediction model.

### 3.5. The Impact of Sugar and Protein in ICM on FP Measurement

Across all treatments, FP decreased proportionally with increased solute concentration, as observed for FP through serial dilution, which is consistent with colligative behavior. However, the magnitude of FP depression differed substantially among sweetener types and between protein-containing and sugar-only matrices. The regression parameters for each system are summarized in Table 6, where the slope of each freezing curve represents the magnitude of FP depression. The lower the slope values, the greater the magnitude of FP depression. Among the sugar-only systems, sucrose produced the steepest depression (slope = −13.33), followed by lactose (−9.85) and corn syrup (−8.82). A similar pattern was also observed in the protein-containing systems, where sucrose produced the greatest FP depression (slope = −17.31), followed by lactose (−13.56) and corn syrup (−10.09). These results align with established literature showing that FP depression is dictated by molality, with lower molecular weight solutes contributing more osmoles per gram [38,39]. Corn syrup, which contains a mixture of glucose, fructose and higher molecular weight oligosaccharides, expresses a weaker colligative effect [24], and therefore depresses FP less efficiently than sucrose or lactose. However, the difference between sucrose and lactose is apparent, and this could be due to the lesser solubility of lactose.

By comparing the protein-containing and sugar-only matrices, it was found that protein amplifies FP depression, but the magnitude of this amplification is not constant, as it varies by sweetener. The difference in slopes between protein-containing and sugar-only systems was largest for lactose (−37.66%), followed by sucrose (−29.86%), and corn syrup (−14.40%). These differences suggest that the degree of protein–sugar interaction in the matrix and their ability to depress FP depend on molecular size, solubility, and hydration behavior of carbohydrates.

These findings indicate that protein–sugar interactions and protein-mediated water structuring contribute measurably to FP behavior. Milk proteins bind significant amounts of water through hydrogen bonding, reducing the amount of freezable water in the system [40]. When sugars are added, they compete with proteins for hydration water, altering the distribution of bound and free water. The removal of water from bulk water increases the effective concentration of the remaining dissolved solutes. A higher solute concentration increases osmotic pressure, which further depresses the freezing point beyond what would be predicted from sugar concentration alone [41,42,43]. The higher FP depression observed with sucrose reflects its stronger water-binding capability, followed by lactose and glucose-based syrup.

The above findings indicate that FP of ICM is influenced not only by the colligative effects of sugar, protein, and other solids in the matrix, but also by the interaction of protein and sugar. This interaction amplifies the magnitude of FP depression by a factor that varies based on the type of sugar, its solubility, molecular weight, and water-binding capacity. In terms of building FTIR models for FP predictions, this factor needs to be addressed, as FTIR models can accurately predict chemical components based on spectra; however, the impact of their interactions on FP is not considered.

## 4. Conclusions and Future Direction

Overall, the model only demonstrated partial feasibility for predicting FP from mid-infrared spectra. The strong cross-validation performance within the testing set indicated that the spectral regions may contain meaningful chemical information related to FP. However, this information was vague, as FP is a physicochemical property derived from the concentration of sugars, salts, stabilizers, and other solids. Therefore, the model generated for this study is not yet capable of predicting FP for ICMs for industrial routine analysis. A greater number of samples are needed to confirm feasibility and robustness for FP models.

Specifically, the predictive capacity of the current PLS model is constrained by two primary limitations: narrow sample composition distribution and the nature of colligative properties in complex matrices. The training dataset was heavily weighted toward the 10% fat level and lacked a balanced representation of 5% and 12% variations, which hindered the model’s ability to map realistic formulation variability. Furthermore, because freezing point is a colligative property rather than a single direct analyte, it is determined by total solute molality and can be altered by matrix interaction. Linear PLS spectral calibrations struggle to track these changes accurately because they do not account for the non-linear, non-additive interactions among ingredients, such as sugars interacting with milk proteins which amplifies freezing point depression. Consequently, expanding this proof-of-concept into a robust industrial tool will require a much larger, compositionally diverse sample set and the evaluation of non-linear modeling algorithms capable of capturing matrix interactions.

## Figures and Tables

**Table 1:** Mayfield ice cream mix samples for freezing point modeling grouped by fat content.

Ice Cream Mix Fat Content (%)	Number of Samples
5	9
10	31
12	3
**Total**	**43**

**Table 2:** Ice cream mix freezing point from the extrapolation method by cryoscopy.

Sample	Freezing Point (m°C)
Publix^®^	−2102
Breyers^®^	−1668
Haagen-Dazs^®^	−2126
Mayfield^®^	−2135
Blue Bell^®^	−2362

**Table 3:** Freezing point of ice cream mixes used for model calibration.

Sample ID	Fat Content (%)	Mean FP (m°C)	Coefficient of Variation (CV, %)
5ecowt (N = 9)	5	−1944	2.65
10.25wt (N = 15)	10	−1969	5.74
10kemps (N = 3)	10	−2160	3.88
10quality (N = 3)	10	−2056	8.83
HMV (N = 9)	10	−1933	8.76
Kayseco (N = 1)	10	−2355	
12wt (N = 3)	12	−1939	0.63

**Table 4:** Components of tested models to predict relationships of quality parameters to FP based on prediction data.

Model	VIFs ^a^							R^2^	AIC ^b^
	Fat	Protein	TS	SNF	Lactose	Sucrose	Sugars		
Full	1424.7	6.51	502.14	572.72	9.4	133.53	44.57	0.318	1586.47
1	1075.4		378.05	482.84	4.22	48.48	25.52	0.319	1585.02
2	970.84		337.68	469.08	4.16	4.37	13.03	0.319	1583.68
3	69.98	5.49	24.04		7.42	121.10	37.17	0.240	1599.21
4	21.56	2.19	5.61		5.07		9.28	0.245	1597.15
5	6.46	1.81			4.22		4.04	0.247	1595.59
6		1.98	1.68		2.9		2.27	0.250	1594.88

^a^ Variance inflation factor; ^b^ akaike information criterion.

**Table 5:** Performance metrics of ice cream mix model.

	RMSEP * (m°C)	Bias (m°C)	Slope	Intercept	R^2^
Training	118.229	−7.029	1.232	449.282	0.269
Cross-validation	119.2	12.268	0.991	−5.190	0.926
Internal validation	104.702	−7.822	0.553	−883.450	0.085
External validation	-	-	-	-	-

* RMSEP: root means square error of prediction.

**Table 6:** Linear regression parameters of ice cream mix matrices.

ICM Matrix	Determined FP of the Undiluted Dispersion (m°C)	Slope	Slope Change (%)
Lactose	−979.17	−9.85	
Sucrose	−1326.55	−13.33	
Corn syrup	−883.99	−8.82	
Protein + lactose	−1344.30	−13.56	−37.66
Protein + sucrose	−1728.84	−17.31	−29.86
Protein + corn syrup	−1004.78	−10.09	−14.40

## Data Availability

The original contributions presented in this study are included in the article. Further inquiries can be directed to the corresponding author.

## Abbreviations

AIC	Akaike information criterion
CV	Coefficient of variation
FP	Freezing point
FTIR	Fourier transform infrared spectroscopy
ICM	Ice cream mix
m°C	Milli degree centigrade
PLS	Partial least squares
RMSEP	Root means square error of prediction
SNF	Solids non-fat
TS	Total solids
VIF	Variance inflation factor
